# High‐dimensional spectral cytometry identifies follicular regulatory CD8^+^ T cells in diffuse large B‐cell lymphoma

**DOI:** 10.1002/cti2.70062

**Published:** 2025-11-03

**Authors:** Alba Díaz Herrero, Phuong‐Ha Le, Loic Renaud, Véronique Meignin, Catherine Thieblemont, Véronique Blanc, Vassili Soumelis, Pierre Tonnerre

**Affiliations:** ^1^ Institut de Recherche Saint‐Louis Université Paris‐Cité, Inserm U1342 Paris France; ^2^ Assistance Publique‐Hôpitaux de Paris, Hôpital Saint‐Louis, Hematology‐Oncologie Université de Paris‐Cité Paris France; ^3^ Pathology Department Hôpital Saint‐Louis, AP‐HP Paris France; ^4^ Institut de Recherche Servier Gif‐sur‐Yvette France

**Keywords:** DLBCL, follicular regulatory CD8^+^ T cells, spectral cytometry, unsupervised analysis

## Abstract

**Objectives:**

Diffuse large B‐cell lymphoma (DLBCL) constitutes 30–40% of non‐Hodgkin lymphoma cases. Despite therapeutic advances, persistence of relapsed cases has been linked to the complex tumor microenvironment (TME) and its interactions with lymphoma cells. In particular, characterising T‐cell subsets, including rare cell types, and their interplay with the remaining TME is crucial for unravelling DLBCL pathogenesis and refining therapeutic strategies.

**Methods:**

Using flow and spectral cytometry with unsupervised analysis, we investigated T‐cell subpopulations across DLBCL biopsies and control lymph nodes (LN). We also inferred communication pathways between T cells and other immune cells in the TME based on the correlation of ligand–receptor expression.

**Results:**

Our analysis revealed a higher frequency of CD8^+^ follicular regulatory T (Tfr) cells in DLBCL biopsies compared to control LN. These cells exhibited an effector‐memory phenotype (CD45RA^−^ CCR7^−^), expressed follicular markers (PD‐1^+^ CXCR5^+^) and had a regulatory profile (CD127^−^ CD25^+^) along with an activation/co‐stimulatory signature (HLA‐DR^+^, ICOS^+^, CD95^+^). Correlation analysis highlighted a co‐stimulatory interaction between lymphoma B cells and CD8^+^ Tfr cells through the ICOS/ICOSL pathway, which may contribute to a protumor effect. Validation in independent scRNAseq and flow cytometry datasets confirmed the notable prevalence of CD8^+^ Tfr cells in DLBCL biopsies.

**Conclusions:**

Our study highlights the utility of high‐dimensional computational cytometry in elucidating T‐cell subpopulations, including an increased frequency of CD8^+^ follicular regulatory T cells and their communication patterns within the DLBCL TME. This unbiased approach sheds light on novel cellular mechanisms in DLBCL, uncovering potential targets and biomarkers for immunotherapy.

## Graphical abstract

In this study, we employed high‐dimensional flow and spectral cytometry with unsupervised analysis to characterise T‐cell subsets in diffuse large B‐cell lymphoma (DLBCL) biopsies compared to control lymph nodes. We identified an increased frequency of CD8^+^ follicular regulatory T cells in DLBCL, a finding validated across independent scRNAseq and flow cytometry datasets, highlighting their consistent presence across cohorts and potential relevance to disease biology.

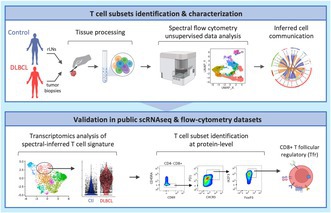

## Introduction

Diffuse large B‐cell lymphoma (DLBCL) is the most common subtype of non‐Hodgkin lymphoma (NHL), characterised by a diffuse pattern of mature B cells.^1^ The standard first‐line treatment R‐CHOP, which includes the anti‐CD20 monoclonal antibody rituximab, has significantly improved patient outcomes over the last two decades. However, 40% of the patients experience relapse or become refractory.^2^ For these patients, advanced therapies such as CAR‐T cell therapy^3^, ^4^, ^5^ and bispecific CD20–CD3 T‐cell engagers (BiTEs)^6^, ^7^, ^8^, ^9^ have become key options in the second‐ and third‐line treatments, respectively. Other strategies, including immune checkpoint inhibition^10^, ^11^, ^12^, ^13^, ^14^ allogeneic stem cell transplantation,^15^, ^16^, ^17^ offer additional hope for long‐term remission. Despite these advances in immunotherapies, significant unmet clinical needs remain for DLBCL patients.^18^

The study of the tumor microenvironment (TME), its cellular components and their complex interactions has become essential to understanding the mechanisms leading to progression and resistance to treatment. Tumor‐infiltrating T cells (TILs) are key players in the TME, contributing to tumor regression in several malignancies.^19^ T cells display high phenotypic and functional diversity in NHL.^20^ In DLBCL TME, T‐cell numbers and subsets vary significantly among patients and have been shown to influence disease progression and patient outcomes.^21^, ^22^, ^23^ Interestingly, despite the abundance of malignant B cells and low immune infiltration,^24^, ^25^ T cells constitute the majority of non‐malignant immune cells in DLBCL tumor samples, further emphasising the important role of T cells in DLBCL pathophysiology.^26^, ^27^ Single‐cell RNA sequencing (scRNA‐seq) analysis of DLBCL has identified four main populations of T cells in the TME: CD4^+^ conventional T helper cells (Th), CD4^+^ T follicular helper cells (Tfh), regulatory T cells (Tregs) and CD8^+^ cytotoxic T lymphocytes (CTL).^27^ In addition, these subsets present different cell states and co‐occurrence patterns in DLBCL, which have been associated with patient prognosis.^23^, ^28^

Traditional methods for T‐cell phenotyping in nodal NHL have relied on immunohistochemistry and flow cytometry, which have provided valuable insights into the T‐cell microenvironment.^24^ These approaches present limitations in achieving deep cellular characterisation because of the limited number of parameters that can be detected, thus requiring a hypothesis‐driven analysis. ScRNA‐seq has emerged as a powerful tool for capturing the heterogeneity of TILs and has become an important component of immune cell deep characterisation studies in NHL.^27^, ^28^, ^29^ Nevertheless, transcriptomic analysis often fails to fully translate mRNA information to protein‐level validation.^30^ In‐depth unsupervised single‐cell analysis of the T‐cell subsets and functional phenotypes, at the protein level, as well as their interactions with other immune cells forming the DLBCL TME, is still needed to elucidate the role of T cells in the pathophysiology of DLBCL.

In this study, we perform a detailed characterisation of DLBCL T cells through unsupervised analysis of high‐dimensional spectral cytometry datasets. Our clustering‐based workflow represents a powerful tool to identify rare T‐cell subsets, and to explore their role in the DLBCL TME by investigating their communication patterns with other immune and malignant cells. Our results, validated in independent external datasets, shed light on the important role of CD8^+^ follicular regulatory T (Tfr) cells, providing a rationale for targeting these cells in future immunotherapy approaches.

## Results

### Supervised flow‐cytometry analysis reveals diverse CD8^+^ T‐cell infiltration in the DLBCL tumor microenvironment

**Figure 1:**
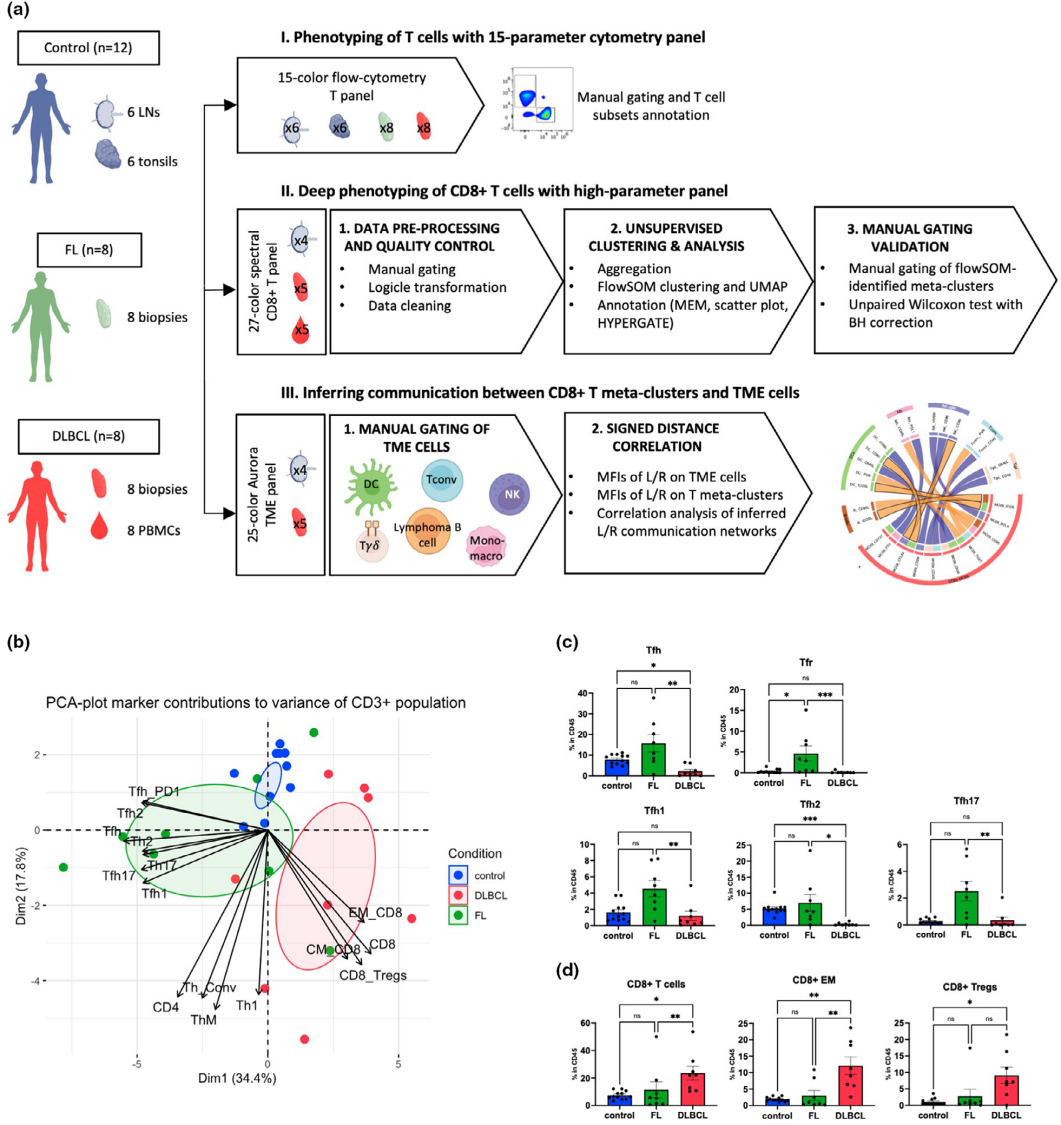
Supervised flow cytometry analysis reveals diverse infiltration of CD8^+^ T cells within the DLBCL tumor microenvironment. **(a)** Workflow for analysing the immunophenotype of T cells in non‐Hodgkin lymphoma using both supervised and unsupervised approaches, and for inferring their interactions with lymphoma B cells and other tumor microenvironment (TME) cells through the signed distance correlation method. **(b)** Principal Component Analysis (PCA) of 22 T‐cell populations across three sample groups; control samples (*n* = 12), FL biopsies (*n* = 8) and nodal DLBCL biopsies (*n* = 8). Top T‐cell contributors to the data variance are displayed with arrows. **(c, d)** Bar plots representing the percentage of specific T‐cell populations in the CD45^+^ gate between control samples (*n* = 12), FL biopsies (*n* = 8) and nodal DLBCL biopsies (*n* = 8) for CD4^+^ (c) and CD8^+^ (d) compartments. Values represent the mean of T‐cell subtype proportion ± sem. Multiple comparisons were performed using Kruskal–Wallis statistical test (not shown) and Dunn's post hoc for pairwise comparisons. ns, not significant; *P‐value < 0.05; **P‐value < 0.01; ***P‐value < 0.001.

We conducted deep T‐cell profiling of the DLBCL TME using three complementary approaches: supervised T‐cell phenotypic characterisation by flow cytometry, unsupervised analysis of high‐parameter spectral cytometry on T‐cell surface markers and inference of T‐cell communication with other cells in the TME. This included a total of DLBCL (*n* = 8), FL (*n* = 8) and control LN/tonsil samples (*n* = 12) (Figure 1a). Because of limited sample availability during the experiment, the unsupervised analysis panel was performed on five DLBCL biopsies and four control lymph node/tonsil samples.

First, we used a combination of 15 T‐cell surface markers to design our gating strategy (Figure 1a, Supplementary figure 1a, Supplementary table 3), which allowed us to identify 22 distinct T‐cell populations (Supplementary figure 1a and b) across the groups. PCA of subset frequencies highlighted group clustering and intra‐group T‐cell homogeneity, with FL clustering driven by Tfh subsets and DLBCL by CD8^+^ T cells (Figure 1b). Tfh subsets appeared significantly higher in FL than in DLBCL, reflecting the well‐known abundance of Tfh in the FL microenvironment.^31^ Moreover, Tregs were more prevalent in both NHL groups, particularly in FL (Figure 1c). No significant difference in the relative abundance of Th memory was observed between groups (Supplementary figure 1c). Interestingly, T‐cell abundance was higher in DLBCL, indicative of T‐cell infiltration (Figure 1c), with CD8^+^ T cell significantly elevated in DLBCL compared to FL and controls (Figure 1d, Supplementary figure 1c).

### Unsupervised clustering reveals three subpopulations of follicular CD8 T cells that are enriched in DLBCL biopsies

**Figure 2:**
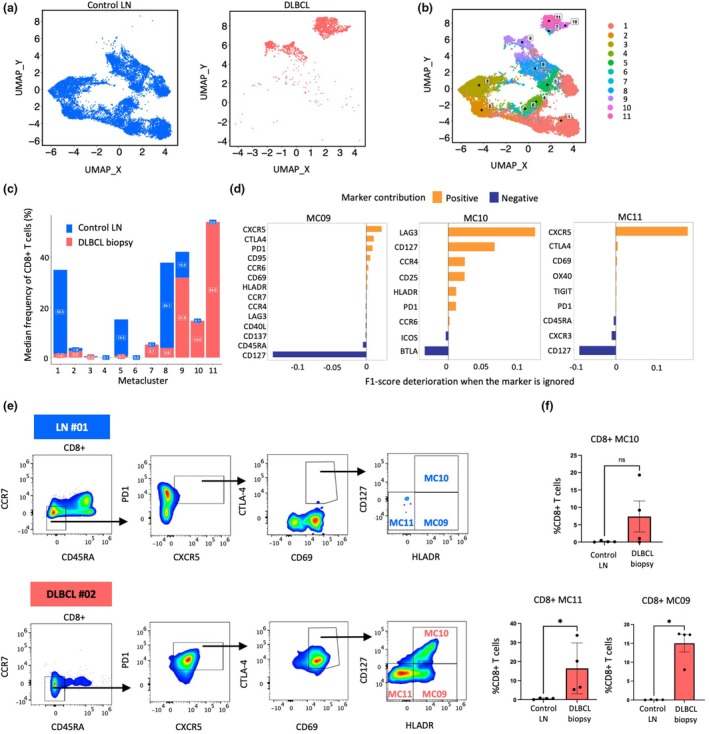
Unsupervised clustering reveals three subpopulations of follicular CD8^+^ T cells that are enriched in DLBCL biopsies. **(a)** UMAP representation of CD8^+^ T cells from LN (*n* = 4) and DLBCL (*n* = 5), divided by the two cohorts. **(b)** UMAP representation of 11 CD8^+^ MCs identified by FlowSOM metaclustering from LN and DLBCL. The data underwent downsampling to the selected number of cells from each sample and markers' MFIs were log‐transformed and z‐scaled before UMAP calculation. **(c)** Stacked bar plot showing the median frequency of 11 MCs within the total CD8^+^ T cells for each sample in control LN (*n* = 4) and DLBCL biopsies (*n* = 5). **(d)** Marker contribution of MC09, MC10 and MC11 was inferred from the deterioration of the F1 score when that marker is ignored, using the HYPERGATE package.^68^ The calculation was performed on the same dataset used for UMAP computation. **(e)** A simplified gating strategy to identify MC09, MC10 and MC11 in a representative control LN and a representative DLBCL biopsy. **(f)** Bar plots representing the frequency (%) of MC09, MC10 and MC11 within CD8^+^ T cell compartment between control LN (*n* = 4) and DLBCL biopsies (*n* = 5). Values are represented as the mean of the MC proportion ± sem. Comparisons between the two cohorts were performed using a two‐tailed Mann–Whitney *U*‐test: ns, not significant; *P < 0.05.

We subsequently employed high‐dimensional spectral cytometric analysis to explore deeper the CD8^+^ T‐cell compartment in DLBCL versus control LN (Figure 1a, Supplementary figure 2). A UMAP representation of the 27‐parameter spectral data highlighted CD8^+^ T‐cell clusters predominantly present in DLBCL patients (Figure 2a). CD8^+^ T cells enriched for CXCR5, PD1, HLA‐DR, CTLA‐4, LAG‐3 and OX40 were identified in DLBCL but were absent in control LN (Supplementary figure 3a). FlowSOM unsupervised clustering further refined the CD8^+^ compartment, identifying 11 unique CD8^+^ MCs (Figure 2b) with their marker expression profiles shown in heatmap (Supplementary figure 3b) and MEM score (Supplementary figure 3c). Among LN‐enriched MCs, MC01 displayed a naïve phenotype (CD45RA^+^ CCR7^+^) (Figure 2c, Supplementary figure 3), confirming the consistency with manual gating (Supplementary figure 1a). MC08, also enriched in LN, expressed CXCR3 (Figure 2c, Supplementary figure 3), a chemokine receptor for interferon‐induced chemokines CXCL9, CXCL10 and CXCL11, with chemotactic effects and roles in T lymphocyte migration, differentiation and function.^32^, ^33^

Frequency quantification revealed enrichment of MC09, MC10 and MC11 in DLBCL CD8^+^ T cells (Figure 2c). These MCs displayed a memory phenotype (CD45RA^−^ CCR7^−^), follicular phenotype (CXCR5^+^ PD1^+^), with high expression of CTLA‐4 and CD69, but differential HLA‐DR and CD127 expressions: MC09 (HLA‐DR^+^ CD127^−^), MC10 (HLA‐DR^+^ CD127^+^) and MC11 (HLA‐DR^−^ CD127^−^) (Figure 2d, Supplementary figure 3c). A manual gating strategy was developed (Figure 2e, Supplementary figure 4a) and confirmed the prevalence of MC09 and MC11, but not MC10, in DLBCL biopsies compared to control LN (Figure 2f) or paired DLBCL PBMCs (Supplementary figure 4b), emphasising the prominence of these two CD8^+^ follicular subsets in the DLBCL TME. Patient DLBCL_3 was excluded from further analysis because of insufficient cell counts, with fewer than 100 CD8^+^ T cells in the final gate (Supplementary table 4).

### Distance correlation of ligand–receptor expressions inferred distinct communication patterns between follicular CD8^+^ T‐cell subsets and lymphoma B cells

**Figure 3:**
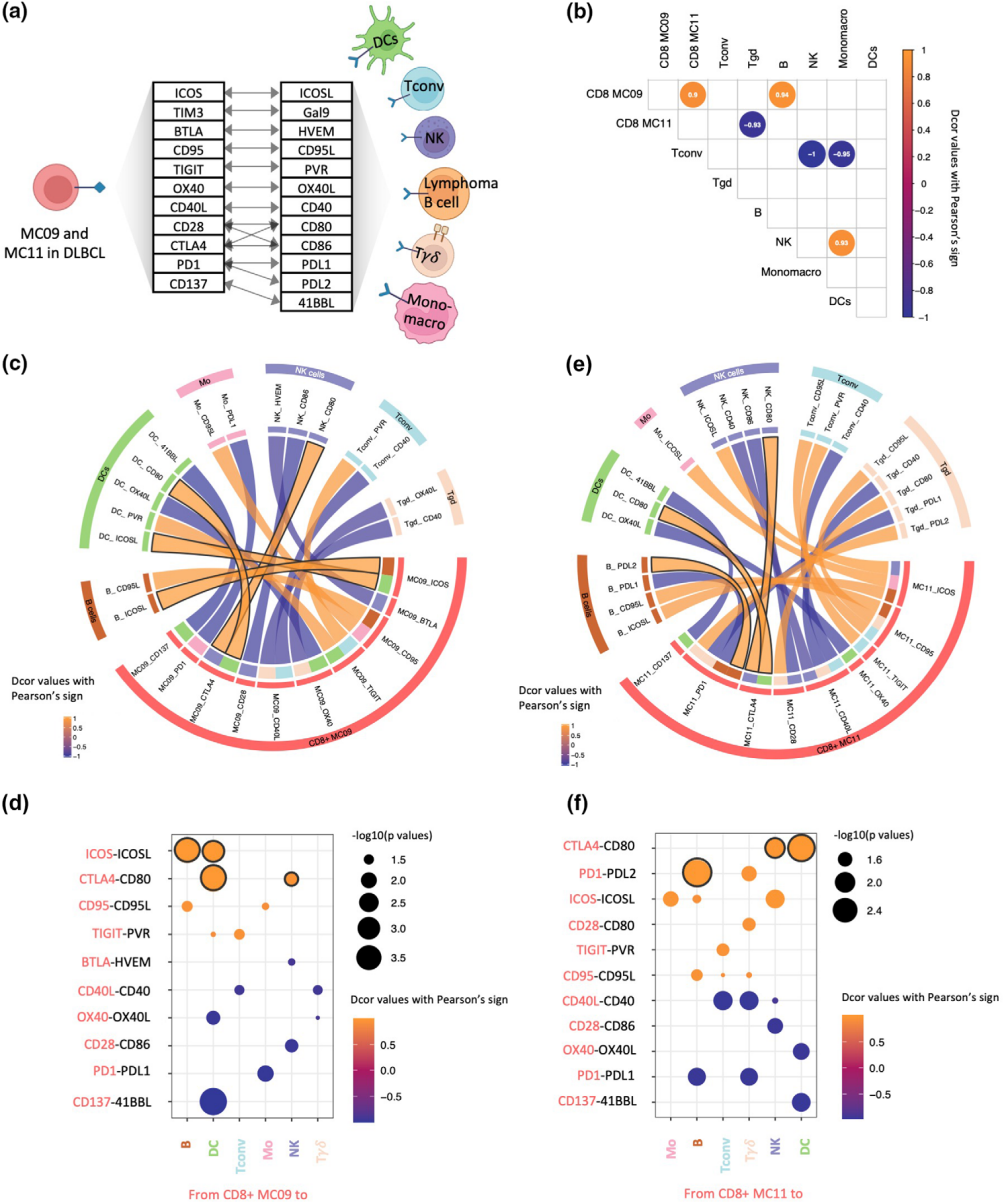
Signed distance correlation of inferred ligand–receptor interactions revealed distinct communication patterns between follicular CD8^+^ T‐cell subsets and lymphoma B cells. **(a)** Schematic representation of the ligand/receptor interactions and cellular communication inference between MC09 and MC11 CD8^+^ T cells and other immune cells from the TME using signed distance correlation analysis. Pairwise distance correlation coefficients were calculated between the median fluorescent intensities (MFIs) of ligand/receptor of CD8^+^ MC09, MC11, and corresponding ligand/receptor of TME cells in DLBCL. The TME cells, including lymphoma B cells (orange), DCs (green), Monomacro (pink), NK cells (violet), conventional T cells (blue), and T𝛾𝛿 (beige) were phenotypically defined using a 25‐colour Aurora panel. MFIs were log‐transformed and z‐normalised before the calculation. **(b)** Correlogram depicting the significant (*P* < 0.05) distance correlations between the frequencies of each MC of interest and TME cell type. Pairwise distance correlation coefficients between the frequency of MCs of interest and the frequency of TME cells in DLBCL were shown as text on each correlation circle. **(c)** Chord diagram of significant (*P* < 0.05) distance correlations between CD8^+^ MC09 and TME cells. Chord's width represents correlation coefficients. Black borders highlight communication axes mentioned in the text. **(d)** Bubble plot of significant (*P* < 0.05) distance correlations between ligand/receptor MFIs on CD8^+^ MC09 and TME cells. Bubble size represents −log_10_(*P*‐value) and colour indicates distance correlation coefficients with sign from Pearson's correlation. Black borders highlight communication axes mentioned in the text. **(e)** Chord diagram of significant (*P* < 0.05) distance correlations between CD8^+^ MC11 and TME cells. Chord's width represents correlation coefficients. Black borders highlight communication axes mentioned in the text. **(f)** Bubble plot of significant (*P* < 0.05) distance correlations between ligand/receptor MFIs on CD8^+^ MC11 and TME cells. Bubble size represents −log_10_(*P*‐value) and colour indicates distance correlation coefficients with sign from Pearson's correlation. Black borders highlight communication axes mentioned in the text.

To understand the functional role of these follicular CD8^+^ subsets, we investigated their potential interactions with lymphoma B cells and other immune cells within the TME. We applied distance correlation to MFIs of ligands and receptors on MC09 and MC11 follicular CD8^+^ T cells and their corresponding ligands and receptors on lymphoma and TME immune cells to infer possible cellular communications (Figure 3a, Supplementary figure 5a). Included cell types were lymphoma B cells, dendritic cells (DCs), conventional T cells (Tconv), natural killer (NK) cells, T gamma delta (T γδ) cells and monocyte–macrophages, all phenotypically defined using a parallel analysis of a 25‐colour spectral panel, performed on the same patient samples (Supplementary figure 5b). We also applied this method to correlate the frequencies of the MCs of interest with those of the TME cells (Supplementary figure 5c, Supplementary table 5). This analysis revealed a significant positive correlation between the frequencies of CD8^+^ MC09 and CD8^+^ MC11, as well as between CD8^+^ MC09 and lymphoma B cells (Figure 3b). Ligand/receptor correlations highlighted potential interactions of MC09 and MC11 with DCs and NK cells via the CTLA4‐CD80 pathway, suggesting an important role of this ligand–receptor pathway in modulating the communication between infiltrating follicular T cells and other immune cells of the TME (Figure 3c and e, Supplementary table 6). In addition, we found potential interaction between HLA‐DR^−^ MC11 T cells and lymphoma B cells through the PD1‐PDL2 axis (Figure 3e and f), suggesting that lymphoma B cells could directly inhibit the activity of MC11 through PD1 signalling. This suggests that HLA‐DR^−^ MC11 cells might be exhausted T cells, which has be linked to poor outcomes.^21^, ^34^, ^35^ Surprisingly, we identified a strong correlation between HLA‐DR^+^ MC09 and lymphoma B cells through the ICOS/ICOSL axis (Figure 3c and d), suggesting a tumor‐supportive role for MC09. This finding emphasises the need for further investigation on the role of HLADR^+^ follicular CD8^+^ T cells in DLBCL.

### Inferring regulatory phenotype of HLA‐DR^+^ MC09 follicular CD8^+^ T cells in DLBCL using scRNA‐seq and flow cytometry public datasets

**Figure 4:**
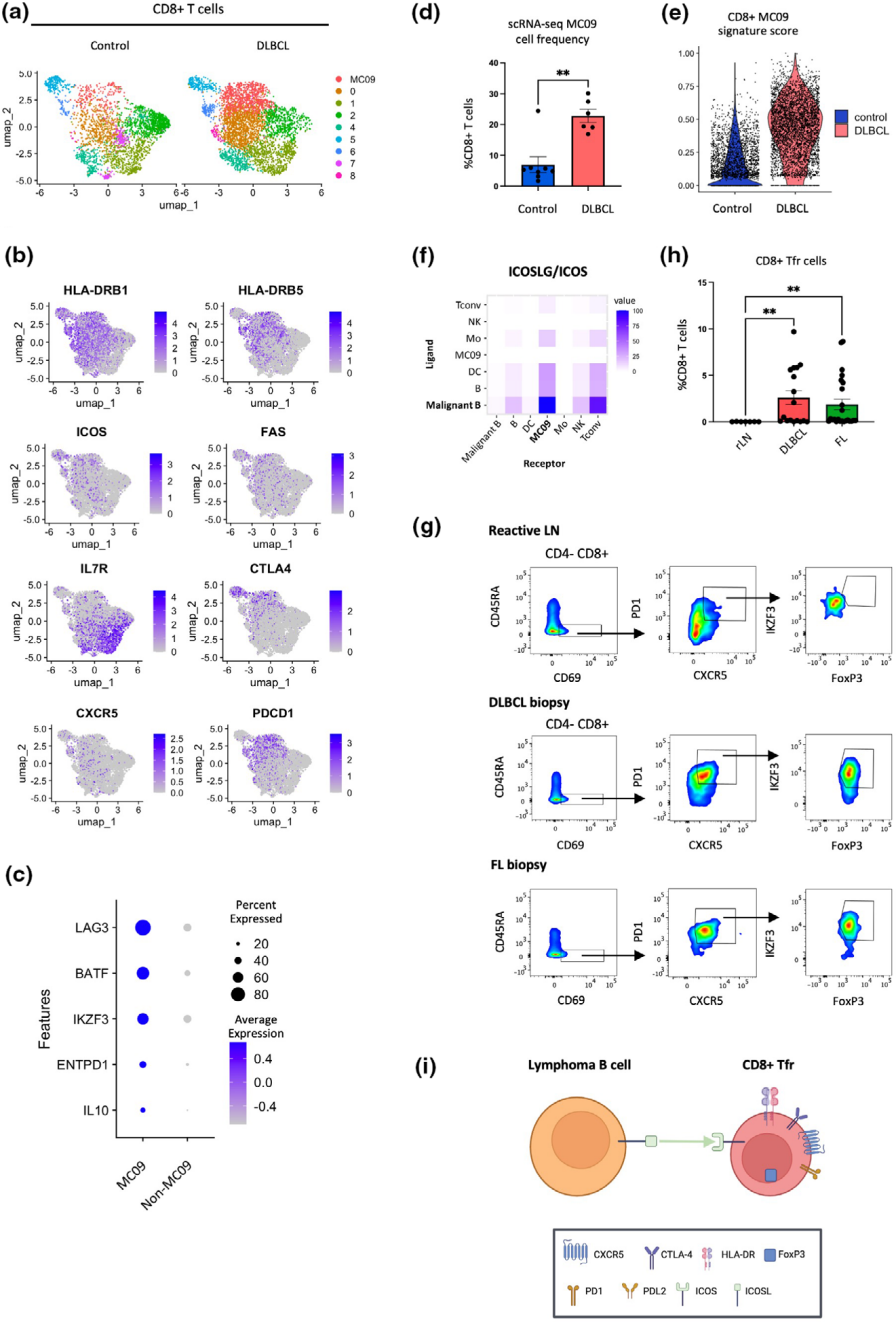
Validation of the regulatory phenotype and function of HLA‐DR^+^ MC09 follicular CD8^+^ T cells in DLBCL using scRNAseq and flow cytometry public datasets. **(a)** Single‐cell RNA sequencing public data clustered. Uniform Manifold Approximation and Projection (UMAP) of tonsils and lymph nodes (control), DLBCL and FL samples. The UMAP show the annotation of MC09 cluster in pink. **(b)** UMAP showing expression of specific genes related to MC09 signature in scRNA‐seq integrated public dataset. **(c)** Dot plot representing expression of differentially expressed genes in MC09 in comparison with all other clusters from **a**. **(d)** Bar plot of MC09 frequency (%) within CD8^+^ T cell compartment between control samples and DLBCL biopsies in scRNA‐seq public data. **(e)** Signature score of genes in **b**, applied to MC09 between control samples and DLBCL scRNA‐seq samples. **(f)** Heatmap of ligand–receptor scores of previously annotated scRNA‐seq cell‐types using the framework ICELLNET (v2). **(g)** Gating strategy to identify CD8^+^ CXCR5^+^ PD1^+^ FoxP3^+^ IKFZ3^+^ Tfr in a reactive LN, a DLBCL biopsy and a FL biopsy. **(h)** Bar plots representing the frequency (%) of CD8^+^ Tfr (gated in **f**) within CD8^+^ T cell compartment between reactive LN (*n* = 7), DLBCL biopsies (*n* = 17) and FL biopsies (*n* = 23). Values are represented as the mean of the MC proportion ± sem. Comparisons between the two cohorts were performed using a two‐tailed Mann–Whitney *U*‐test: ns, not significant; **P < 0.01. **(i)** Schematic depicting potential interaction between CD8^+^ Tfr cells and lymphoma B cells.

To further characterise the phenotype and function of HLA‐DR^+^ MC09 follicular CD8^+^ T cells, we conducted unsupervised clustering and cell‐type annotation on integrated public scRNA‐seq datasets from Steen *et al*.,^36^ Roider *et al*.^27^ and an internal dataset, including DLBCL samples (*n* = 6), LN samples (*n* = 4) and tonsil samples (*n* = 4) (Figure 4a, Supplementary figure 6c, Supplementary table 7). This analysis identified different clusters of CD8^+^ T cells, including one transcriptionally similar to MC09 (CTLA4^+^ CXCR5^+^ PD1^+^ HLA‐DR^+^ CD127^−^ ICOS^+^) (Figure 4b). Interestingly, this cluster also expressed LAG3, BATF, IKFZ3, ENTPD1 and IL‐10, markers typical of regulatory T cells (Figure 4c). MC09 cells were significantly over‐represented in DLBCL compared to LN and tonsil controls (Figure 4d, Supplementary figure 5, Supplementary table 7). The MC09 spectral signature score (HLA‐DRB1, HLA‐DRB5, FAS, LAG3, ICOS, CXCR5, PDCD1, CTLA4) was highly enriched in DLBCL (Figure 4e), and pathway enrichment suggests a regulatory function for MC09 (Supplementary figure 6b, Supplementary table 8). Interestingly, the cell–cell communication analysis based on ligand–receptor interactions reveals higher communication scores for the axis ICOS/ICOSL of MC09 and malignant B cells compared to other cell types (Figure 4f, Supplementary figure 6d). Moreover, the axis CD80‐CTLA4 presents high communication scores for MC09 with DCs and monocytes–macrophages (Supplementary figure 6e).

Building on our scRNA‐seq findings, we investigated the expression of the Treg markers FoxP3 and IKFZ3 in follicular CD8^+^ T cells using flow cytometry data from Roider *et al*.^20^ This dataset included seven tumor‐free reactive LNs (rLNs), 17 DLBCL and 24 FL tissue samples. Focussing on CD8^+^ T memory follicular cells (CD45RA^−^ CD69^+^ CXCR5^+^ PD1^+^), we observed substantial co‐expression of FoxP3 and the induced‐Treg marker IKZF3^37^ in both DLBCL and FL samples (Figure 4g). These CD8^+^ CD45RA^−^ CD69^+^ CXCR5^+^ PD1^+^ FoxP3^+^ IKZF3^+^ cells, which we annotated as follicular regulatory CD8^+^ T cells (CD8^+^ Tfr), were significantly enriched in DLBCL and FL compared to rLNs (Figure 4h). Importantly, this represents the first description of CD8^+^ Tfr cells in DLBCL, and to our knowledge, also in FL. In contrast, CD4^+^ Tfr cells, identified using the same gating strategy, were significantly enriched only in FL, as previously reported.^20^ Together, these findings highlight a previously unrecognised enrichment of CD8^+^ Tfr cells in DLBCL, underscoring a potentially distinct immunoregulatory niche within the TME.

## Discussion

By building an in‐depth supervised and unsupervised analysis pipeline of cytometry data with distance correlation, our study enabled deep phenotyping of rare cell types in DLBCL and effectively inferred their communication with other cells in the TME of DLBCL at the protein level. Compared to the TME of FL, characterised by CD4^+^ Tfh infiltration, the DLBCL TME showed a significant presence of CD8^+^ T cells, which are known to be mainly exhausted, contributing to the immunosuppressive TME.^20^, ^34^ Our analysis suggests that these exhausted T cells could correspond to CD8^+^ MC11, which exhibit a less active HLA‐DR^−^ phenotype and potentially interact with lymphoma B cells through the PD1‐PDL2 axis.

Unsupervised clustering, combined with validation using public datasets, has revealed a novel subset of CD8^+^ Tfr‐infiltrating DLBCL TME. Given that HLA‐DR expression is often downregulated in DLBCL and linked to a poorer prognosis,^38^, ^39^, ^40^ the enrichment of HLADR^+^ CD8^+^ Tfr cells offers a unique insight into the regulatory landscape of the DLBCL TME. In fact, HLA‐DR expression was associated with active Tregs rather than resting ones, and HLA‐DR^+^ Tregs are more suppressive than their HLA‐DR^−^ counterparts both in health and in disease.^41^, ^42^ *In vitro* studies showed that CD8^+^HLA‐DR^+^ Tregs exhibit similar functional characteristics to CD4^+^FOXP3^+^ Tregs, in suppressing the activity of stimulated autologous CD8^+^ effector T cells.^43^, ^44^, ^45^ Moreover, lenalidomide, an IKZF1/3 degrader, combined with R‐CHOP, improves the poor prognosis of A7 DLBCL patients by boosting T‐cell trafficking and MHC expression, promoting CD8^+^ T‐cell recognition of tumor cells.^46^ We suggest that the IKZF3‐enriched CD8^+^ Tfr cells identified in our study could be cellular targets of lenalidomide. Overall, these findings support the regulatory profile of CD8^+^ Tfr cells infiltrating the TME in DLBCL.

Tregs exert their immunosuppressive effects through multiple mechanisms, including the secretion of immunosuppressive cytokines IL‐10; consumption of IL‐2; production of immunosuppressive adenosine by ectoenzymes CD39; modulation of antigen‐presenting cell activity via CTLA4, LAG3, and ICOS, while BATF is crucial for their homeostasis and stability in preventing autoimmune pathology.^47^, ^48^, ^49^, ^50^, ^51^ In this study, CD8^+^ Tfr cells showed increased expression of CTLA4, IL‐10, CD39, LAG3, ICOS and BATF, suggesting that these suppressive mechanisms may contribute to their enhanced suppressive capacity.

Cell–cell communication is often inferred from gene expression, but proteomics, with its direct protein abundance measurement, is preferable for these analyses.^52^ In this project, we employed a novel signed distance correlation method^53^, ^54^ on spectral cytometry data to capture both linear and nonlinear ligand–receptor expression correlation in DLBCL. Our correlation analysis inferred 20 probable cell–cell interactions between follicular CD8^+^ T‐cell subsets and other immune cells in the DLBCL TME. Our data suggested that CD8^+^ Tfr cells might engage with lymphoma B cells and DCs via ICOS/ICOSL interactions, potentially stabilising FOXP3 function but also increasing IL‐10 production by Tregs, enhancing suppressive potency.^55^, ^56^ Moreover, CD25^high^CD127^low/neg^ Tregs, including activated ICOS^+^ Tregs, can suppress conventional T cells, with their generation linked to ICOS/ICOSL interactions with lymphoma B cells.^57^

In addition, our interaction analysis suggests that CD8^+^ Tfr cells may exert their suppressive function on cytotoxic CD8^+^ T cells primarily by overexpressing CTLA‐4, potentially sequestering CD80 or CD86 ligands on DCs away from CD28.^58^ CTLA‐4 signalling may play multifaceted roles by either promoting the suppressive function of CD8^+^ Tfr cells or enhancing the inhibitory signals on exhausted T cells.^58^, ^59^ ICOS/ICOSL and CTLA‐4/CD80 interactions are further corroborated by communication analysis on external scRNA dataset, supporting our protein‐level findings using spectral flow cytometry. Altogether, these molecular and cellular mechanisms may contribute to an immunosuppressive environment that facilitates the growth of lymphoma B cells (Figure 4i).

The identification of CD8^+^ Tfr cells in both DLBCL and FL highlights a previously unrecognised component of the tumor immune microenvironment. Unlike CD4^+^ Tfr cells, which are predominantly enriched in FL^20^, CD8^+^ Tfr cells appear to play an important role in both lymphoma subtypes, potentially contributing to immune suppression within germinal center‐like microenvironments.

Nevertheless, *in situ* validation of cell–cell interactions and functional studies is still required to confirm the role of CD8^+^ Tfr cells within the DLBCL TME. Additionally, a larger cohort is needed to evaluate the potential association between CD8^+^ Tfr cells infiltration and patient survival outcomes, as well as their correlation with clinical characteristics.

Altogether, by leveraging technological advances in high‐dimensional cytometry and scRNA‐seq, our study expands on existing knowledge of lymphoma‐infiltrating T cells and infers their potential roles in shaping the immunosuppressive TME in DLBCL. Specifically, we highlight the prevalence of CD8^+^ Tfr cells in the TME of DLBCL. These findings pave the way for further investigations into their functional mechanisms, association to clinics and potential as a therapeutic target in DLBCL.

## Methods

### Sample collection and processing

Diffuse large B‐cell lymphoma biopsy samples (*n* = 8) and follicular lymphoma (FL) samples (*n* = 8) were collected from patients at the Hemato‐Oncology department of the Hospital Saint Louis (Paris, France). DLBCL diagnosis was performed by expert haematopathologists following the diagnostic criteria established by previous pathologic descriptions of PMBL from the literature and international classifications^60^, ^61^ with the support of the French Lymphopath network.^62^ Clinical and demographic information of the DLBCL patients is provided in Supplementary table 1 and Supplementary table 2, with the samples comprising both diagnostic and relapsed cases. Non‐metastatic tissue controls (*n* = 12), including lymph node (LN) samples (*n* = 6) and tonsil samples (*n* = 6), were collected from patients at the hospital Bichat and Robert Debré (Paris, France). This study was approved by the CLEA‐2020‐113 ethical committee and institutional review board. All individuals included in this study signed an informed consent. Set‐up experiments used frozen PBMCs from healthy donors from *Etablissement Francais du Sang of Saint Louis Hospital* (EFS, Paris).

DLBCL biopsies and LN samples underwent mechanical dissociation the same day of collection, before being filtered through a 70‐mm‐pore Strainer, washed three times and resuspended in RPMI 1640 + Glutamax medium (Life technologies, Carlsbad, USA; Cat# 61870‐010) supplemented with 10% FBS, 1% MEM NEAA (Life Technologies; Cat# 11140‐050), 1% Sodium Pyruvate (Life Technologies; Cat# 11360) and 1% Penicillin/Streptomycin (Life Technologies; Cat# 15140‐122). The single‐cell suspensions were rested overnight at 37°C with 5% CO_2_ in a humidified atmosphere.

### Panel design and optimisation for high‐dimensional immunophenotyping

Two antibody panels were designed: one targeting T‐cell biology and the other characterising additional immune cells in the TME (Supplementary table 3). Markers were chosen based on immune lineage, activation and differentiation status, chemokine receptors and checkpoints implicated in T‐cell biology with clinical relevance in NHL. Similarity indices between pairs of fluorochromes were considered acceptable if they were below 0.9. Fluorochrome assignments considered fluorochromes' brightness and markers' expression levels, prioritising brighter fluorochromes for low‐expression markers.

Multiple step staining protocols were tested, dividing antibodies into 2–5 staining steps based on expression levels. Staining resolution was optimised by evaluating fold‐change differences in fluorescence intensity and spillover between single stains and fluorochrome mixes. Protocols were selected for minimal spillover and least alteration of fluorescence intensity of the lowest number of antibodies. Optimisation was performed on frozen PBMCs from healthy donors. Cells were analysed on a Cytek Aurora 5L Spectral Analyzer (Cytek Bioscience, Fremont, USA). Analysis of panel optimisation was performed using SpectroFlo v3.0 (Cytek Bioscience) and FlowJo v10.8.1 (BD Biosciences, Franklin Lakes, USA).

### Flow cytometry

Fresh cell suspensions were resuspended to 2 × 10^6^ cells mL^−1^ in DPBS1X (Life Technologies Cat# 14190‐094) and incubated with LIVE/DEAD blue (Biolegend, San Diego, USA; Cat# 423101) for 15 min and washed twice with DPBS1X (Life Technologies; Cat# 14190‐094). Cells were incubated with CXCR5 antibody (R&D Systems, Minneapolis, USA) for 30 min at 37°C, washed, followed by an antibody mix (CCR7, CD4, PD1, CD25, CCR4, CCR6, CD20, CD45RA, CD8, CD127, CXCR3, CD45 and CD3) for 10 min at 4°C in 100 μL of FACS buffer (DPBS1X+ EDTA 2 mM) (Invitrogen, Waltham, USA; Cat# 15575‐038) + 1% Human Serum (Sigma, Burlington, USA; Cat# 4522‐100). Antibody technical information was provided in Supplementary table 3. Cells were washed and fixed with FFPE 1%. Cell suspensions were washed and resuspended in 200 μL of FACS buffer and analysed with a LRS Fortessa cytometer (BD). Cell viability was assessed by the Zombie Aqua staining and T‐cell subsets were gated using FlowJo v10.8.1 (BD Biosciences).

### Spectral cytometry

DLBCL and LN samples were resuspended in DPBS1X (Life Technologies; Cat# 14190‐094) to 2 × 10^6^ cell mL^−1^ and incubated with LIVE/DEAD blue (Thermofisher, Waltham, USA; Cat# L23105) for 15 min at room temperature. After washing, cells were divided for T cell and TME panel staining. For the T cell panel, cells were incubated for 30 min at 37°C with chemokine receptor antibodies (CXCR3, CXCR5, CCR4, CCR6, CCR7, CD25 and CD127) in 100 μL DPBS1X with 2 mM EDTA (Invitrogen; Cat# 15575‐038) and 1% of Human Serum (Sigma; Cat# 4522‐100). Suspensions were washed twice with DPBS1X and incubated with OX40, 4‐1BB, CTLA‐4, CD40L, CD20, LAG‐3, BTLA, ICOS, PD1, CD28, CD3, Tim‐3, CD4, CD45, CD45RA, CD69, Fas, HLA‐DR and TIGIT in 100 μL of Brilliant Buffer (BD; Cat# 566349) for 30 min at room temperature. Antibodies information was provided in Supplementary table 3.

For the TME panel, cells were stained with anti‐TCR γδ for 10 min at room temperature in 100 μL of DPBS1X complemented with 2 mM EDTA and 1% Human Serum. After washing with DPBS1X, cells were incubated in 100 μL of Brilliant Buffer (BD; Cat# 566349) with an antibody cocktail (CD10, CD11c, CD123, 4‐1BBL, CD14, PVR, FasL, CD19, CD1c, CD20, CD21, OX40L, HVEM, PD‐L1, ICOSL, NKp46, CLEC9A, CD40, CD80, CD86, Galectin‐9, HLA‐DR and PDL2) for 30 min at room temperature.

Then, all suspensions were washed and fixed with PFA 1% (Alfa Aesar, Ward Hill, USA; Cat# J19943). Cells were analysed on a Cytek Aurora 5L Spectral Analyzer (Cytek Bioscience). Spectral data were unmixed in SpectroFlo (Cytek Biosciences), with healthy PBMCs, and beads (BD Biosciences, Cat# 51‐90‐9001229 and 51‐90‐9001291) as single stain references and unstained cells of each individual sample for autofluorescence extraction. Each sample underwent spillover correction using SpectroFlo (Cytek Biosciences).

### Data pre‐processing and quality control

Data were saved as Flow Cytometry Standard (FCS) 3.0 files and subsequently imported into FlowJo software. Subsequently, manual pregating using the FlowJo software version 9 (BD, San Jose, CA) was performed to remove aggregates and dead cells and to keep only live and single cells. For the T cell panel, two populations were analysed: CD3^+^ T cells, and CD3^+^ CD4^−^ CD8^+^ T cells. In parallel, the TME panel included six manually gated populations: gamma delta T cells, conventional CD3^+^ T cells, CD19^+^ B cells, natural killer (NK) cells, dendritic cells (DCs), and monocytes/macrophages. Data from these cell types across all eight samples were imported in RStudio (version 4.2.3) for preprocessing and quality control. This included applying logicle transformation using the *flowCore* R package^63^ with fixed parameters (w = 0.5, t = 10 000, m = 2, a = 0), and automatically removing outlier events caused by abnormal flow behaviours such as clogs and other technical issues using the *flowCut* R package.^64^

### Unsupervised clustering and analysis

Cell populations were identified by FlowSOM,^65^ one of the best‐performing unsupervised clustering techniques identified in the benchmark of Weber *et al*. (2016).^66^ FlowSOM uses a Self‐Organising Map (SOM) to cluster similar cells into finely defined cell types, which are subsequently grouped into metaclusters (coarse‐grained cell types) (MCs). Clustering was performed on the CD8^+^ T cell population using the FlowSOM R package. A combined dataset of 75 875 cells, sampled from the CD8^+^ populations of four control LNs and five DLBCL biopsies, was generated for analysis (Supplementary table 4). The FlowSOM model in this study was created using a grid of 10 × 10 (100 clusters), grouping into 11 CD8^+^ MCs using the following markers: CXCR5, HLADR, CD127, TIGIT, CD45RA, CD28, OX40, CD137, CCR7, ICOS, CD95, CCR4, CD25, PD1, CD69, TIM3, LAG3, CXCR3, BTLA, CD40L, CTLA4 and CCR6. The number of MCs was determined using FlowSOM, guided by the elbow method^65^ and the final selection was made by the authors to balance granularity with biological robustness. The resulting clusters were visualised using Uniform Manifold Approximation and Projection (UMAP) dimensionality reduction method. Annotation of these clusters was performed based on their Marker Enrichment Modelling (MEM) scores.^67^ Additionally, the HYPERGATE package^68^ was employed to automatically gate the clusters identified by FlowSOM. This package evaluates the quality of the automatic gating strategy by measuring the decrease in F1‐score of a cluster (the F1 score ranges from 0 to 1, with 1 indicating perfect reproduction of the corresponding FlowSOM‐identified MC) when a parameter/marker is omitted from the gating strategy. The lower the F1‐score, the more important the parameter/marker is.

### Signed distance correlation to infer cellular communication

The correlation between logical‐transformed median fluorescence intensities (MFIs) of T cells and TME cells, gated in FlowJo from both LN and DLBCL biopsies, was determined using Distance correlation^69^ via the platform Signed Distance Correlation (SiDCo).^70^ Distance correlation allows for the examination of both linear and nonlinear correlations, providing insights into direct and indirect feature correlations. The sign of the distance correlation is given by the sign of the Pearson correlation following Pardo‐Diaz *et al*.^53^ Distance correlation values represent the strength of the correlation between pairs of markers. Only correlations with *P*‐value < 0.05, which were considered significant, are visualised in chord diagrams and bubble heatmaps.

### Single‐cell RNA sequencing data analysis

ScRNA‐seq data analysis from Roider *et al*.,^27^ Steen *et al*.^28^ and an internally generated dataset, was conducted on R (v4.3.2) using the package Seurat (v4.1.1).^71^ The first 50 principal components (PC) were used to integrate the data using the package Harmony^72^ (v0.1.0). scRNA‐seq data were filtered to retain cells with more than 200 genes and less than 10% of mitochondrial genes, normalised and log_2_‐transformed. Dimensionality reduction was performed with principal components analysis (PCA). The Louvain clustering algorithm^73^ was used for spot clustering, applied to a shared nearest‐neighbour (SNN) graph. Differentially expressed genes (DEG) were identified using a threshold of |log_2_FC| higher than 0.25 and a *P*‐value below 0.05 from the Wilcoxon rank‐sum test. Clusters were manually annotated based on DEG. Malignant B cells were annotated based on their chromosomal copy number variation with the R package CopyKAT (v1.0.5).^74^ Functional pathway enrichment on the top 50 genes was performed using Metascape.^75^ Module scores (https://satijalab.org/seurat/reference/addmodulescore) were applied to compare relative expression of meta clusters' gene signatures between sample types. Ligand–receptor interactions between cell types were scored using the framework ICELLNET (v2).^76^

### Manual gating validation

To validate the existence and frequency of the MCs identified by FlowSOM, FlowJo was used as an independent validation tool. Frequencies were calculated based on the percentage of cells assigned to each MC among all CD8^+^ T cells in each sample. We utilised an optimised manual gating strategy, guided by the MEM score and HYPERGATE F1‐score. This strategy aimed to replicate the marker expression profiles of the population of interest. We validated the phenotype of the population of interest using an external flow‐cytometry dataset from Roider *et al*.,^20^ including a Treg panel (Viability, CD3, CD4, CD8, CD45RA, CD25, CD69, ICOS, CXCR5, PD1, FoxP3, IKZF3, Ki67).

### Statistical analysis

Quantitative variables of high‐dimensional cytometry were compared using the Wilcoxon rank‐sum test. Data are represented as individual values, medians, and the interquartile range of the median. Statistical analyses were performed using Prism 9.0 (GraphPadSoftware Inc., La Jolla, USA). *P*‐values were corrected for multiple testing using the Benjamini–Hochberg (BH) correction method.

## Author contributions

**Alba Díaz Herrero:** Conceptualization; data curation; formal analysis; funding acquisition; investigation; methodology; project administration; resources; software; validation; visualization; writing – original draft; writing – review and editing. **Phuong‐Ha Le:** Data curation; formal analysis; methodology; software; validation; visualization; writing – original draft; writing – review and editing. **Loic Renaud:** Resources. **Véronique Meignin:** Resources. **Catherine Thieblemont:** Conceptualization; resources. **Véronique Blanc:** Conceptualization; funding acquisition; project administration. **Vassili Soumelis:** Conceptualization; funding acquisition; project administration. **Pierre Tonnerre:** Conceptualization; funding acquisition; project administration; supervision; visualization; writing – review and editing.

## Conflict of interest

A.D.H. was supported by a PhD fellowship from Servier. V.B. is a Servier employee. V.S. is currently employed by Owkin. The remaining authors declare no competing interests.

## Supporting information


Supplementary figure 1
Supplementary figure 2
Supplementary figure 3
Supplementary figure 4
Supplementary figure 5
Supplementary figure 6
Supplementary table 1
Supplementary table 2
Supplementary table 3
Supplementary table 4
Supplementary table 5
Supplementary table 6
Supplementary table 7
Supplementary table 8

## Data Availability

The scRNA‐seq dataset was retrieved from heiDATA under accession code VRJUNV and from GEO under the accession number GSE182436. Roider *et al*.^20^ flow cytometry data is available at https://doi.org/10.6084/m9.figshare.24915633. All the analysis scripts have been uploaded to https://github.com/a‐diaz‐herrero/DLBCL_spectral_cytometry. The remaining data supporting the findings of this study are available from the corresponding authors upon request.
