# Supplementary material for: High‐dimensional spectral cytometry identifies follicular regulatory CD8+ T cells in diffuse large B‐cell lymphoma
Source: Clin Transl Immunology. 2025 Nov 3;14(11):e70062. doi: 10.1002/cti2.70062 (PMC12581182; doi:10.1002/cti2.70062)
Supplement: Supplementary file 1 — Supplementary figure 1 Supplementary figure 2 Supplementary figure 3 Supplementary figure 4 Supplementary figure 5 Supplementary figure 6 Supplementary table 1 Supplementary table 2 Supplementary table 3 Supplementary table 4 Supplementary table 5 Supplementary table 6 Supplementary table 7 Supplementary table 8 [file CTI2-14-e70062-s001.pdf]

Supplementary Figures and Tables

High-dimensional spectral cytometry identifies follicular regulatory CD8$^+$ T cells in Diffuse large B cell lymphoma

Alba Díaz Herrero*, Phuong-Ha Le*, Loic Renaud, Véronique Meignin, Catherine Thieblemont, Véronique Blanc, Vassili Soumelis, Pierre Tonnerre

* These authors contributed equally to this work

(a)

T cell subtype	Immunophenotype
**Parent**	Lymphocytes, single cells, LIVE DEAD - CD45+ CD20- CD3+
**CD4+**	CD4+
**Tfh**	CD4+ CXCR5+
**Tfh1**	CD4+ CXCR5+ CXCR3+ CCR6-
**Tfh2**	CD4+ CXCR5+ CXCR3- CCR6-
**Tfh17**	CD4+ CXCR5+ CXCR3- CCR6+
**Tregs**	CD4+ CD25+ CD127-
**Tfr-like**	CD4+ CD25+ CD127- CXCR5+
**Th Conv**	CD4+ CD25- CD127+/-
**Th Naïve**	CD4+ CD25- CD127+/- CD45RA+ CCR7+
**ThM**	CD4+ CD25- CD127+/- CD45RA+/- CCR7+/-
**Th1**	**ThM**: CXCR3+ CCR4- CCR6-
**Th1/Th17**	**ThM**: CXCR3+ CCR4- CCR6+
**Th2**	**ThM**: CXCR3- CCR4+ CCR6-
**Th17**	**ThM**: CXCR3- CCR4+ CCR6+
**CD8+**	CD8+
**CD8+ CM**	CD8+ CD45RA- CCR7+
**CD8+ EM**	CD8+ CD45RA- CCR7-
**CD8+ EMRA**	CD8+ CD45RA+ CCR7-
**CD8+ Naïve**	CD8+ CD45RA+ CCR7+
**CD8+ PD1+**	CD8+ CCR7- PD1+
**CD8+ CXCR5+**	CD8+ CXCR5+
**CD8+ Tregs**	CD8+ CD127- CD25+

(b)

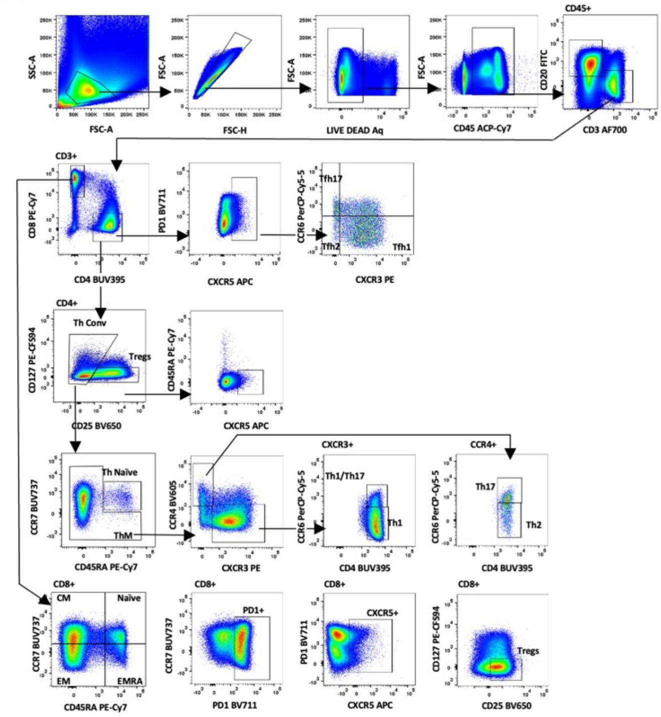

(c)

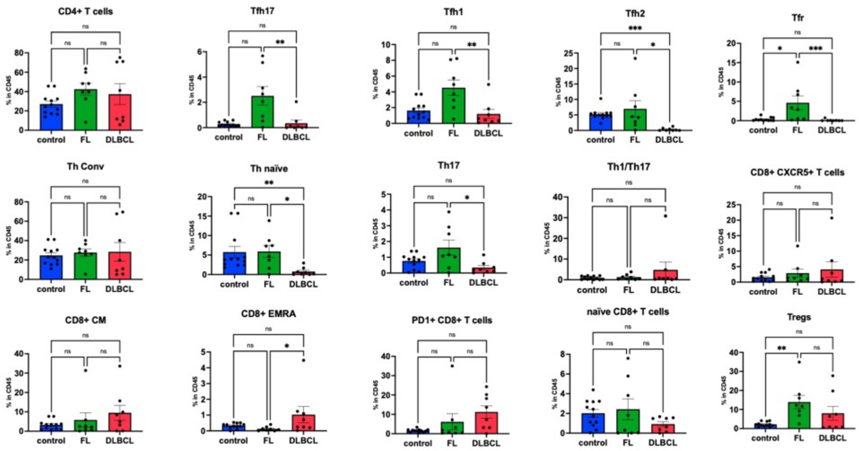

Supplementary figure 1. Gating strategy and T cell subset frequencies in DLBCL and FL TME

(a) Marker combination to identify 22 T cells populations: CD4$^+$ T cells, follicular T helper cells (Tfh), follicular T helper 1 cells (Tfh1), follicular T helper 2 cells (Tfh2), follicular T helper 17 cells (Tfh17), regulatory T cells (Tregs), follicular regulatory T cells (Tfr), Conventional T helper cells (Th Conv), T helper Naïve cells (Th Naïve), T helper Memory cells (ThM), T helper 1 cells (Th1), T helper 1/17 cells (Th1/17), T helper 2 cells (Th2), T helper 17 cells (Th17), CD8$^+$ T cells, CD8$^+$ Central Memory T cells (CD8$^+$ CM), CD8$^+$ Effector Memory T cells (CD8$^+$ EM), CD8$^+$ CD45$^+$ Effector Memory T cells (CD8$^+$ EMRA), CD8$^+$ Naïve T cells (CD8$^+$ Naïve), CD8$^+$ PD1$^+$ T cells, CD8$^+$ CXCR5$^+$ T cells, CD8$^+$ regulatory T cells (CD8$^+$ Tregs).

(b) Manual gating strategy on a representative DLBCL sample. Arrows are used to visualize the relationships between gates.

(c) Bar plots representing the percentage of specific T cell populations in the CD45$^+$ gate. Values represent the mean of T cell-subtype proportion ±SEM. Multiple comparisons were performed using Kruskal-Wallis statistical test (not shown) and Dunn's post-hoc for pairwise comparisons. ns, not significant; $*p < 0.05$; $**p < 0.01$.

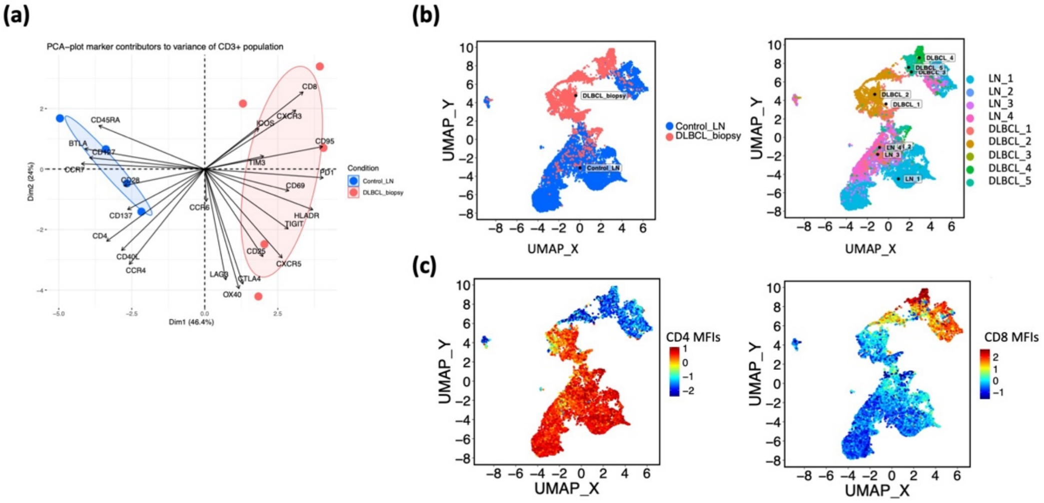

Supplementary figure 2. High dimensional immune phenotyping of CD3$^+$ T cells reveals differences between control LN and DLBCL biopsies.

- **(a)** PCA plot of marker contributors to variance of CD3$^+$ T cell population. All markers' MFIs were logicle-transformed and z-scaled before PCA calculation.
- **(b)** UMAP plots depict CD3$^+$ T cells colored by cohort (left) and donors (right). Prior to UMAP calculation, the data underwent downsampling to the selected number of cells from each sample and all markers' MFIs were logicle-transformed and z-scaled.
- **(c)** UMAP plots depict CD3$^+$ T cells colored by CD4 expression (left) and CD8 expression (right). Prior to UMAP calculation, the data underwent downsampling to the selected number of cells from each sample and all markers' MFIs were logicle-transformed and z-scaled.

(a)

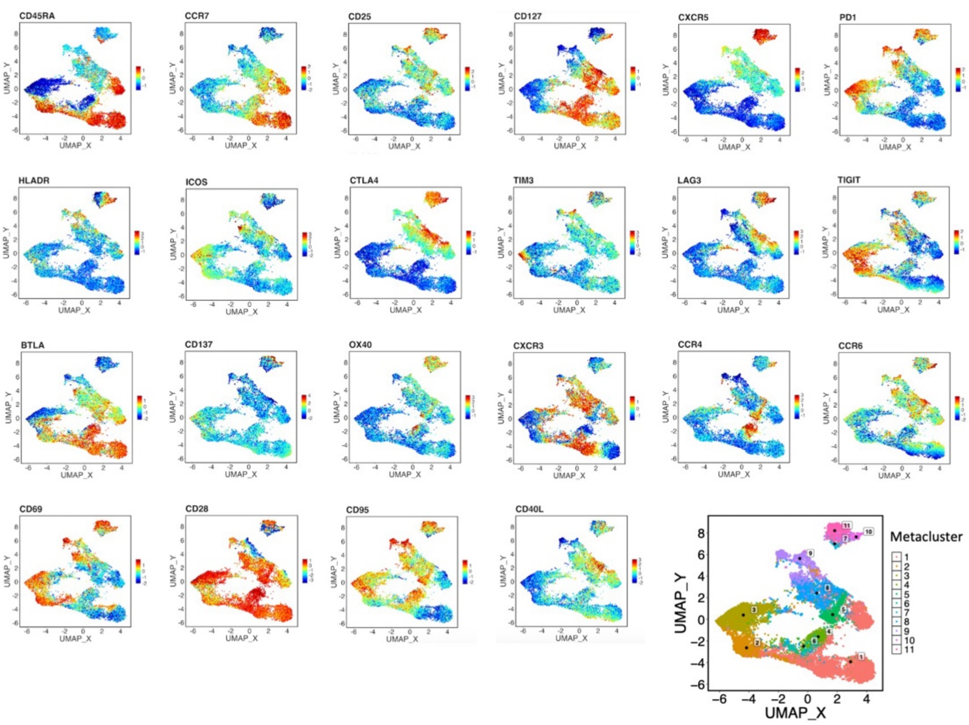

(b)

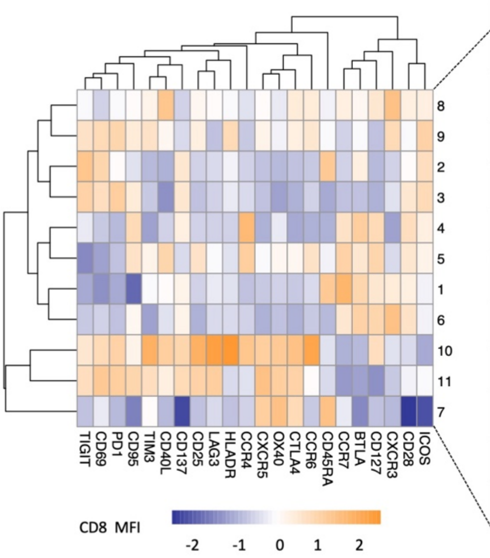

(c)

MC	MEM score
8	▲ CXCR3+6 CXCR5+2 CD127+2 CTLA4+2 CD95+1 PD1+1 CD40L+1 CCR6+1 ▼ CD45RA-6 CCR7-2 BTLA-2 CD69-1
9	▲ CD69+3 CXCR3+3 CTLA4+3 CXCR5+2 PD1+2 HLADR+1 TIGIT+1 ICOS+1 CD95+1 CCR6+1 ▼ CD45RA-4 CD127-3 CCR7-2 CD28-1 LAG3-1 BTLA-1
2	▲ CD45RA+6 TIGIT+4 CD69+3 ICOS+1 PD1+1 ▼ CD127-5 CTLA4-3 CCR7-2 CXCR3-2 CXCR5-1 CD28-1 CD95-1 CCR4-1 LAG3-1 BTLA-1 CCR6-1
3	▲ TIGIT+2 PD1+2 CD69+2 ICOS+1 CD95+1 ▼ CD45RA-9 CD127-4 CTLA4-3 CCR7-2 BTLA-2 CXCR5-1 LAG3-1 CXCR3-1 CD40L-1
4	▲ CD127+5 CCR4+3 BTLA+2 TIGIT+1 CD28+1 CCR7+1 CD95+1 ▼ CD45RA-9 CXCR3-5 CTLA4-3 PD1-2 CXCR5-1 CD69-1 TIM3-1 LAG3-1 CCR6-1
5	▲ CD127+5 CXCR5+2 CCR7+2 CCR4+2 CTLA4+2 CD95+1 CD25+1 BTLA+1 CCR6+1 ▼ CD45RA-5 CXCR3-4 TIGIT-3 CD69-3 PD1-2
1	▲ CD45RA+7 CD127+6 BTLA+3 CCR7+2 LAG3+1 CXCR3+1 ▼ TIGIT-4 PD1-3 CD69-3 CXCR5-2 CCR4-2 CTLA4-2 ICOS-1 CD95-1 CCR6-1
6	▲ CXCR3+6 CD127+5 BTLA+2 CCR7+1 CD95+1 ▼ CD45RA-9 CTLA4-3 CXCR5-1 TIGIT-1 PD1-1 TIM3-1 LAG3-1
10	▲ CTLA4+5 CD127+4 LAG3+4 CXCR5+3 HLADR+3 PD1+3 CD69+3 TIGIT+2 CCR4+2 CCR6+2 OX40+1 CD95+1 CD25+1 TIM3+1 ▼ CD45RA-9 CXCR3-4 BTLA-3 CCR7-2 CD28-1 ICOS-1
11	▲ CXCR5+4 CD69+4 CTLA4+4 PD1+3 TIGIT+2 LAG3+2 OX40+1 CD95+1 CCR4+1 CD25+1 ▼ CD45RA-9 CD127-5 CXCR3-4 BTLA-3 CCR7-2 CD28-1
7	▲ CD45RA+10 CXCR5+4 CTLA4+4 OX40+1 ▼ CD127-3 CD28-3 CXCR3-3 BTLA-3 TIGIT-1 CD137-1 CCR7-1 ICOS-1 CD95-1 CCR4-1

Supplementary figure 3. Marker expression profiles and associated MEM score of 11 CD8⁺ MCs

(a) UMAP of marker expression profile of CD8⁺ T cells from LN (n=4) and DLBCL biopsies (n=5). The data underwent down sampling to the selected number of cells from each sample and markers' MFIs were logicle-transformed and z-scaled before UMAP calculation.

(b) MFIs heatmap with column-scaled z-scored MFIs.

(c) MEM score of 11 CD8⁺ MCs.

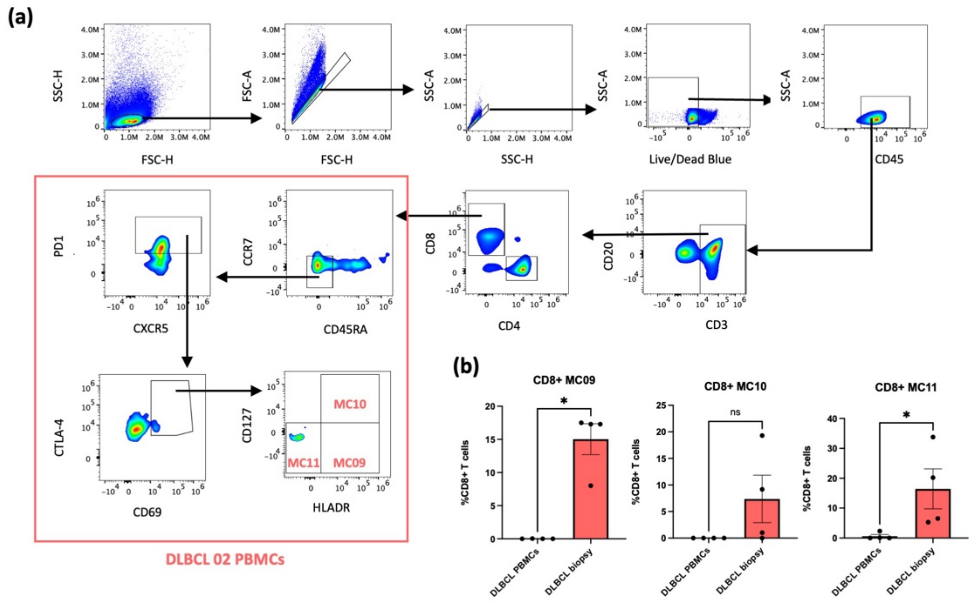

Supplementary figure 4. Follicular CD8$^+$ MC09 and MC11 are more abundant in the DLBCL TME compared to PBMCs.

(a) Gating strategy of CD8$^+$ MC09, MC10 and MC11 on a representative DLBCL PBMCs sample.

(b) Bar plots representing the frequency (%) of MC09, MC10 and MC11 within CD8$^+$ T cell compartment between DLBCL PBMCs (n=4) and DLBCL biopsies (n=4). Values are represented as the mean of the MC proportion ± SEM. Comparisons between the two cohorts were performed using a two-tailed Mann-Whitney statistical test: ns, not significant; $*p < 0.05$; $**p < 0.01$.

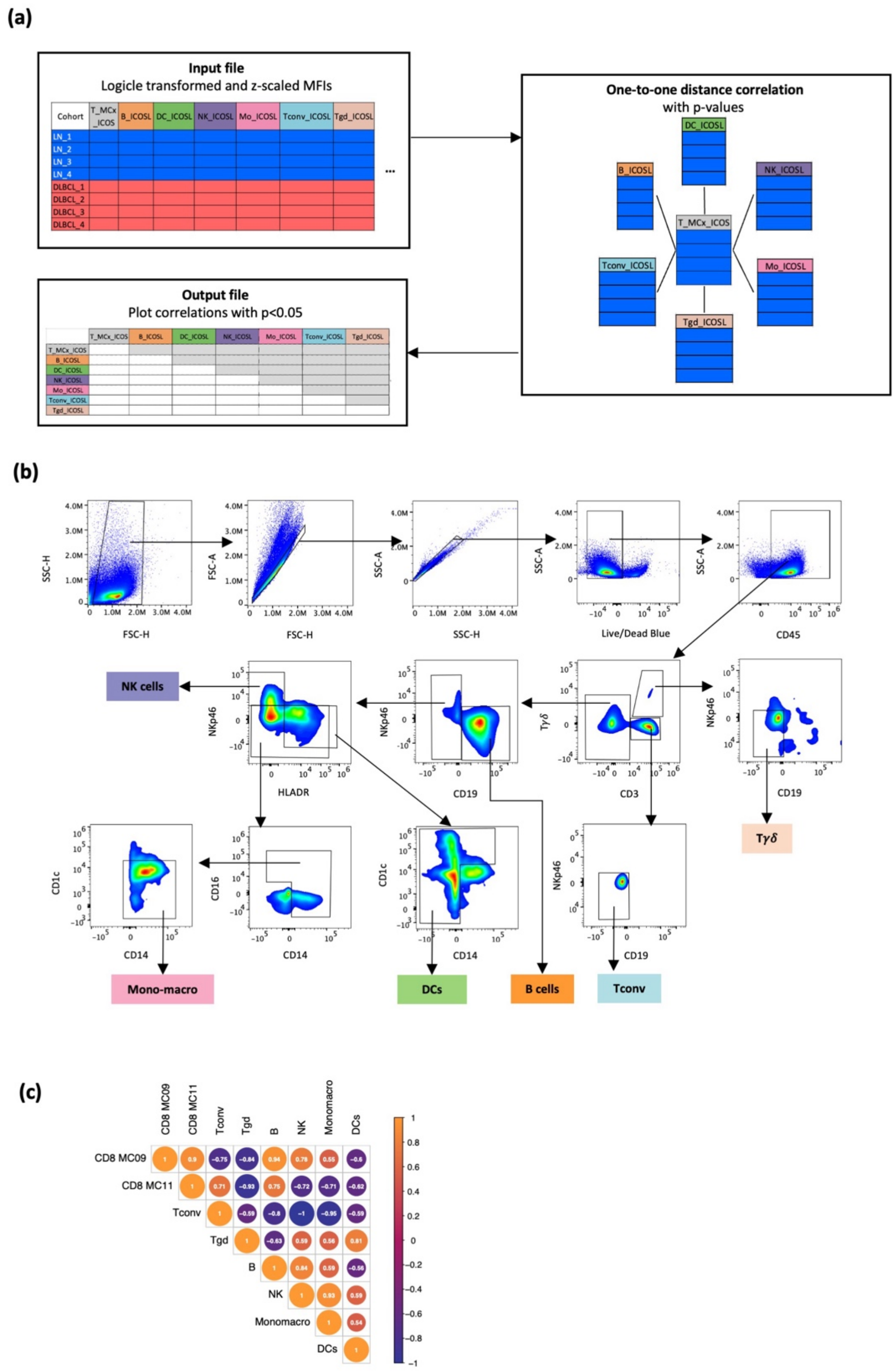

Supplementary figure 5. Signed distance correlation inferred cellular communication between CD8$^+$ follicular subpopulations and other TME cells in DLBCL biopsies.

(a) Workflow of signed distance correlation computation.

(b) Gating strategy of TME panel on a representative sample.

(c) Correlogram depicting all the distance correlations between the frequencies of each MC of interest and TME cell type. Pairwise distance correlation coefficients were calculated between the frequency of MCs of interest and the frequency of TME cells in DLBCL, shown as text on each correlation circle.

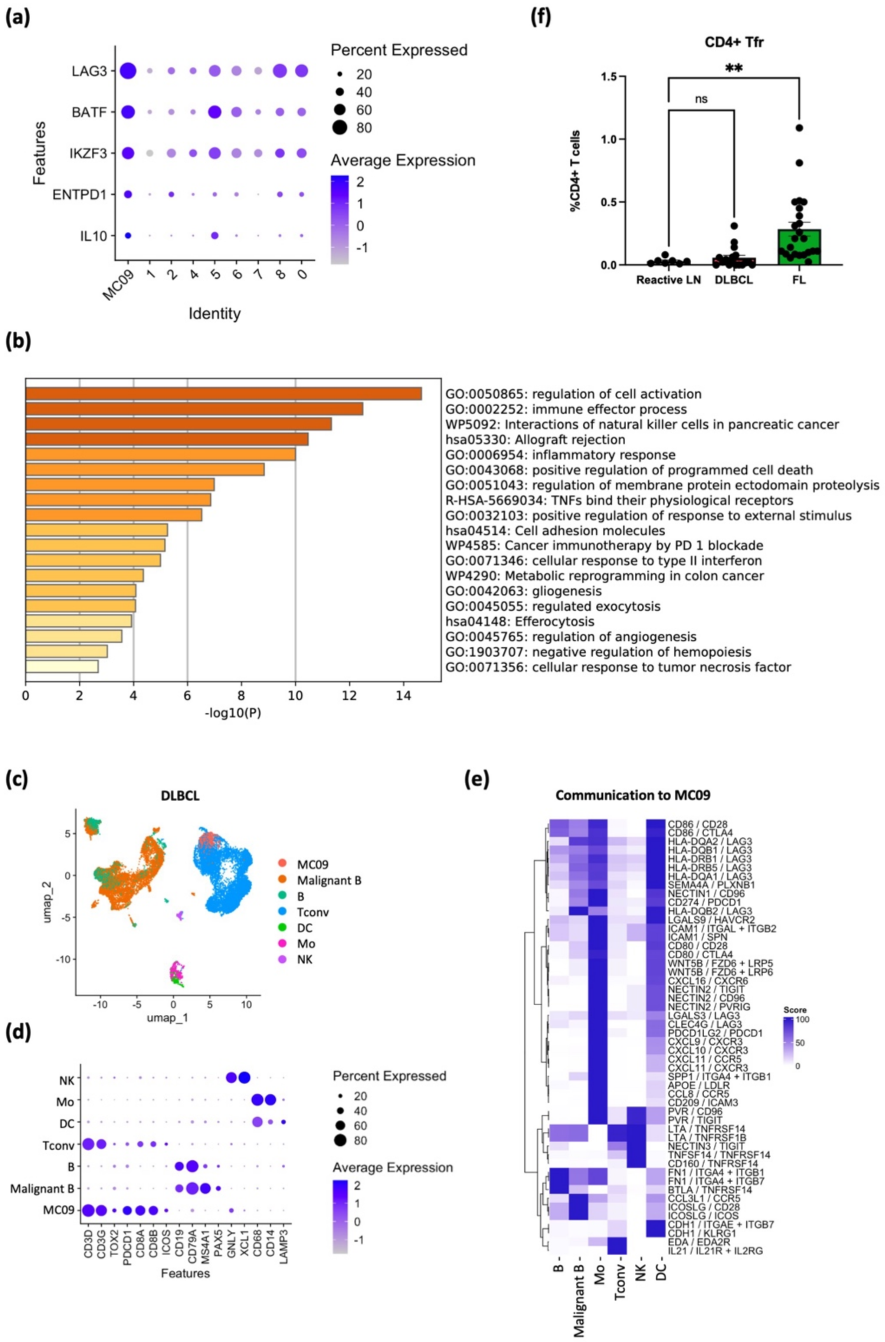

Supplementary figure 6. scRNA-seq analysis of public datasets suggests regulatory profile of CD8$^+$ Tfr cells in the TME of DLBCL.

(a) Dot plot representing expression of differentially expressed genes (DEGs) in MC09 and each of the clusters from Figure 4A.

(b) Pathway enrichment analysis from integrated scRNA-seq datasets based on the top 50 DEGs of CD8+ Tfr.

(c) UMAP of cells identified by unsupervised clustering on integrated scRNA-seq datasets, including DLBCL samples (n=6), LN samples (n=4) and tonsil samples (n=4).

(d) Dot plot representing differential gene expression of annotated cell types.

(e) Heatmap of cell-cell communication analysis by ICELLNET based on ligand- receptor interactions.

(f) Bar plots representing the frequency (%) of CD4$^+$ Tfr (identified using a gating strategy similar to that for CD8$^+$ Tfr in Figure 4F) within CD4$^+$ T cell compartment between reactive LN (n = 7), DLBCL biopsies (n = 17) and FL biopsies (n=23). Values are represented as the mean of the MC proportion ± SEM. Comparisons between the two cohorts were performed using a two-tailed Mann-Whitney *U*-test: ns, not significant; $* P < 0.05$; $** P < 0.01$.

Supplementary table 1. Clinical characteristics of patients.

patient_ID	age	sex	diagnosis_inclusion	stage	COO	time_biopsy	IPI	Supervised flow cytometry	Unsupervised spectral	TME-COM
DLBCL_6	58	F	DLBCL	2	nonGC	relapse	1	yes		
DLBCL_1	56	F	DLBCL	4	GC	diagnosis	2	yes	yes	yes
DLBCL_2	73	F	DLBCL	4	nonGC	diagnosis	5	yes	yes	yes
DLBCL_7	51	M	DLBCL	3	nonGC	diagnosis	2	yes		
DLBCL_8	65	F	DLBCL	4	GC	diagnosis	3	yes		
DLBCL_3	78	M	DLBCL	4	nonGC	relapse	5	yes	yes*	
DLBCL_4	66	M	DLBCL	4	GC	relapse	4	yes	yes	yes
DLBCL_5	36	F	DLBCL	4	nonGC	relapse	4	yes	yes	yes
FL_1	52	M	FL	3a		diagnosis		yes		
FL_2	64	M	FL	4		diagnosis		yes		
FL_3	51	M	FL	3		relapse		yes		
FL_4	60	F	FL	4		relapse		yes		
FL_5	49	M	FL	4		diagnosis		yes		
FL_6		F	FL			diagnosis		yes		
FL_7		F	FL			diagnosis		yes		
FL_8	70	M	FL	2		diagnosis		yes		

* Sample DLBCL_3 (p25) was excluded from downstream analyses (manual gating validation and distance correlation) due to insufficient CD8+ T-cell count, see Supplementary Table 4.

Supplementary table 2. Demographics of patients.

Variables	N patients	Percentage
**Age**		
Age ≤60 years	3	60
Age >60 years	2	40
**Sex**		
Female	3	60
Male	2	40
**Stage**		
I-II	0	0
III	0	0
IV	5	100
**Cell of Origin**		
GCB	2	40
non-GCB	3	60
**Relapse status at diagnosis**		
non-relapse	2	40
relapse	3	60
**IPI**		
2	1	20
4	2	40
5	2	40

Supplementary table 3. Marker and fluorochrome information for each experiment

Experiment	Fluorophore / Tag	Marker	Vendor	Catalog number	Clone	Dilution
Spectral cytometry T cell panel	BUV737	CD197 (CCR7)	BD	749676	2-L1-A	1:200
	BUV615	CD134 (OX40)	BD	751320	L106	1:20
	BUV805	CD4	BD	612888	SK3	1:500
	APC-Cy7	CD45	BD	557833	2D1	1:50
	BUV661	CD137 (41BB)	BD	741642	4B4-1	1:50
	APC	CD185 (CXCR5)	R&D systems	FAB190A-025	51505	1:50
	BUV395	CD45RA	BD	740315	5H9	200
	BB700	TIGIT	BD	747846	741182	1:200
	BV650	CD25	biolegend	302634	BC96	1:80
	BUV563	CD28	BD	741392	CD28.2	1:200
	BV480	CD95 (Fas)	BD	746675	DX2	1:50
	BV421	CD278 (ICOS)	BD	562901	DX29	1:50
	BV711	CD279 (PD1)	biolegend	329927	EH12.2H7	1:100
	BV750	CD69	BD	747522	FN50	1:20
	APC-R700	CD127	BD	565185	HIL-7R-M2	1:500
	APC-Fire810	HLADR	biolegend	307673	L243	1:500
	BV605	CD194 (CCR4)	Biolegend	359418	L291H4	1:100
	BUV496	CD3	BD	564810	UCHT1	1:200
	PE	CD183 (CXCR3)	BD	557185	1C6/CXCR3	1:100
	PE-CF594	CD272 (BTLA)	BD	564801	J168-540	1:50
	BV785	CD366 (Tim-3)	Biolegend	345032	F38-2E2	1:20
	PECy5	CD154 (CD40L)	Biolegend	310808	24-31	1:20
	PeCy7	CD152 (CTLA-4)	Biolegend	349913	L3D10	1:20
	PerCP/Cyanine5.5	CD196 (CCR6)	Biolegend	353406	G034E3	1:20
	FITC	CD223 (LAG-3)	Enzo	ALX-804-806F-C100	17B4	1:40
	Pacific Orange	CD20	Thermofisher	MHCD2030	HI47	1:1000
	LIVE DEAD blue	viability	Thermofisher	L34961		1:1000
Spectral cytometry TME panel	BUV661	TCR γδ	BD	750019	11F2	1:200
	BUV563	CD19	BD	741361	HIB19	1:1000
	AF700	Galectin-9	R&D systems	FAB20453N	1015214	1:100
	APC	CD275 (ICOSL)	R&D systems	FAB165A	136726	1:20
	BV510	CD40	BD	563456	5C3	1:100
	PerCP/Cyanine5.5	CD137L (41BBL)	Biolegend	311517	5F4	1:20
	BV650	CD123	biolegend	306020	6H6	1:50
	AF647	CD335 (NKp46)	biolegend	331909	9E2	1:500
	BB700	CD270 (HVEM)	BD	746034	CW10	1:50
	BV711	CD10	biolegend	312226	HI10a	1:200
	BV421	CD252 (OX40L)	BD	563766	ik-1	1:20
	APC-Fire810	HLADR	biolegend	307674	L243	1:500
	BV605	CD80	BD	563315	L307.4	1:50
	BV785	PDL2 (CD273)	BD	563843	MIH18	1:40
	BUV395	CD178 (FasL)	BD	744103	NOK-1	1:50
	BUV737	CD155 (PVR)	BD	748586	SKII.4	1:100
	PE-CF594	CD86	BD	562390	331 (FUN-1	1:80
	PECy5	CD21	BD	551064	B-ly4	1:200
	PE	CD370 (CLEC9A)	Biolegend	353803	8F9	1:100
	PeCy7	CD1c(BDCA1)	Biolegend	331516	L161	1:300
	Spark Blue 550	CD14	Biolegend	367148	63D3	1:1000
	eFluor450	CD11c	Thermofisher	48-0116-42	3.9	1:200
	Pacific Orange	CD20	Thermofisher	MHCD2030	HI47	1:1000
	PerCP-eFluor 710	CD274 (PD-L1)	Thermofisher	46-5983-42	MIH1	1:80
	LIVE DEAD blue	viability	Thermofisher	L34961		1:1000
Flow cytometry	BUV737	CD197 (CCR7)	BD	749676	2-L1-A	1:200
	BUV395	CD4	BD	564724	RPA-T4	1:40
	APC-Cy7	CD45	BD	557833	2D1	1:50
	APC	CD185 (CXCR5)	R&D systems	FAB190A-025	51505	1:50
	BV650	CD25	biolegend	302634	BC96	1:80
	BV711	CD279 (PD1)	biolegend	329927	EH12.2H7	1:100
	BV605	CD194 (CCR4)	Biolegend	359418	L291H4	1:100
	PE	CD183 (CXCR3)	BD	557185	1C6/CXCR3	1:100
	PerCP/Cyanine5.5	CD196 (CCR6)	Biolegend	353406	G034E3	1:20
	PE-CF594	CD127	BD	562396	HIL-7R-M20	1:10
	FITC	CD20	BD	65	556655	1:60
	AF700	CD3	Biolegend	300423	UCHT0	1:80
	PE-Cy7	CD45RA	BD	560675	HI100	1:160
	Pe-Cy5	CD8	BD	555636	HIT8a	1:50
	LIVE DEAD Aqua Zombie	viability		423101		1:500

Supplementary table 4. Number of manually gated cells of each sample.

Patient_ID	Sample_ID	Cohort	Number of CD3+ cells	Number of CD8+ cells
LN06	LN_1	Control LN	316647	45007
LN08	LN_2	Control LN	50874	11197
LN09	LN_3	Control LN	69788	9328
LN10	LN_4	Control LN	24284	3857
p03	DLBCL_1	DLBCL biopsy	1612	446
p07	DLBCL_2	DLBCL biopsy	42691	5124
p25	DLBCL_3	DLBCL biopsy	82	44*
p27	DLBCL_4	DLBCL biopsy	742	474
p35	DLBCL_5	DLBCL biopsy	702	398

* Sample DLBCL_3 (p25) was excluded from downstream analyses (manual gating validation and distance correlation) due to insufficient CD8+ T-cell count.

Supplementary table 5. Distance-correlation coefficients (signed by Pearson's *ρ*) for cell-frequency comparisons between DLBCL TME populations, CD8$^+$ MC09 and MC11. Corresponding *P*-values are shown in parentheses.

	CD8 MC09	CD8 MC11	Tconv	Tgd	B	NK	Monomacro	DCs
CD8 MC09	1 (1)	0,903 (0,048)	-0,751 (0.125)	-0,839 (0.081)	0,943 (0.028)	0,783 (0.109)	0,554 (0.223)	-0,601 (0,199)
CD8 MC11		1 (1)	0,714 (0,143)	-0,925 (0.037)	0,747 (0.127)	-0,718 (0.141)	-0,708 (0.146)	-0,616 (0,192)
Tconv			1 (1)	-0,589 (0.205)	-0,802 (0.099)	-0,998 (0.001)	-0,948 (0.026)	-0,587 (0,206)
Tgd				1 (1)	-0,634 (0.183)	0,589 (0.206)	0,564 (0.218)	0,808 (0,096)
B					1 (1)	0,842 (0.079)	0,591 (0.205)	-0,557 (0,221)
NK						1 (1)	0,930 (0.035)	0,586 (0,207)
Monomacro							1 (1)	0,536 (0,232)
DCs								1 (1)

Supplementary table 6. Distance-correlation coefficients and their corresponding *P*-values for ligand and receptor MFIs across DLBCL TME populations, $CD8^+$ MC09, and $CD8^+$ MC11.

TME_LR	T_LR	dcor_values	dcor_p_values
NK_ HVEM	CD8_MC09_BTLA	-0,915811906	0,042094047
DC_ 41BBL	CD8_MC09_CD137	-0,999783482	0,000108259
NK_ CD86	CD8_MC09_CD28	-0,96394586	0,01802707
Tconv_ CD40	CD8_MC09_CD40L	-0,936145643	0,031927178
Tgd_ CD40	CD8_MC09_CD40L	-0,934105821	0,032947089
B_ CD95L	CD8_MC09_CD95	0,943944946	0,028027527
Mo_ CD95L	CD8_MC09_CD95	0,915724694	0,042137653
DC_ CD80	CD8_MC09_CTLA4	0,999579137	0,000210432
NK_ CD80	CD8_MC09_CTLA4	0,959770695	0,020114653
B_ ICOSL	CD8_MC09_ICOS	0,999207513	0,000396243
DC_ ICOSL	CD8_MC09_ICOS	0,996205914	0,001897043
DC_ OX40L	CD8_MC09_OX40	-0,968644011	0,015677995
Tgd_ OX40L	CD8_MC09_OX40	-0,905258459	0,04737077
Mo_ PDL1	CD8_MC09_PD1	-0,982261063	0,008869468
DC_ PVR	CD8_MC09_TIGIT	0,906695849	0,046652076
Tconv_ PVR	CD8_MC09_TIGIT	0,94202907	0,028985465
DC_ 41BBL	CD8_MC11_DLBCL_CD137	-0,972331013	0,013834494
NK_ CD86	CD8_MC11_DLBCL_CD28	-0,956831947	0,021584026
Tgd_ CD80	CD8_MC11_DLBCL_CD28	0,941493854	0,029253073
NK_ CD40	CD8_MC11_DLBCL_CD40L	-0,908897429	0,045551285
Tconv_ CD40	CD8_MC11_DLBCL_CD40L	-0,974683774	0,012658113
Tgd_ CD40	CD8_MC11_DLBCL_CD40L	-0,973272831	0,013363584
B_ CD95L	CD8_MC11_DLBCL_CD95	0,932299345	0,033850328
Tconv_ CD95L	CD8_MC11_DLBCL_CD95	0,906822469	0,046588765
Tgd_ CD95L	CD8_MC11_DLBCL_CD95	0,908252326	0,045873837
DC_ CD80	CD8_MC11_DLBCL_CTLA4	0,993574446	0,003212777
NK_ CD80	CD8_MC11_DLBCL_CTLA4	0,980095373	0,009952314
B_ ICOSL	CD8_MC11_DLBCL_ICOS	0,917555874	0,041222063
Mo_ ICOSL	CD8_MC11_DLBCL_ICOS	0,95639983	0,021800085
NK_ ICOSL	CD8_MC11_DLBCL_ICOS	0,97377529	0,013112355
DC_ OX40L	CD8_MC11_DLBCL_OX40	-0,962619871	0,018690065
B_ PDL1	CD8_MC11_DLBCL_PD1	-0,969074279	0,01546286
B_ PDL2	CD8_MC11_DLBCL_PD1	0,995687385	0,002156308
Tgd_ PDL1	CD8_MC11_DLBCL_PD1	-0,967493756	0,016253122
Tgd_ PDL2	CD8_MC11_DLBCL_PD1	0,953965933	0,023017033
Tconv_ PVR	CD8_MC11_DLBCL_TIGIT	0,939629878	0,030185061

Supplementary table 7. Information of scRNA-seq samples from public datasets.

sample_ID_article origin	sample ID	tissue	dataset	number of cells (post QC)	number of CD8+ cells	number of MC09 cells	Frequency of MC09 in CD8+ T cells
DLBCL002	sc_DLBCL1	DLBCL	Steen et al	5524	621	105	16,91
DLBCL007	sc_DLBCL2	DLBCL	Steen et al	1434	220	52	23,64
DLBCL008	sc_DLBCL3	DLBCL	Steen et al	3000	1209	332	27,46
DLBCL111	sc_DLBCL4	DLBCL	Steen et al	4400	1819	353	19,41
DLBCL2	sc_DLBCL5	DLBCL	Roider et al	5224	68	13	19,12
DLBCL3	sc_DLBCL6	DLBCL	Roider et al	2627	73	22	30,14
rLN1	sc_LN1	LN	Roider et al	3089	434	106	24,42
rLN2	sc_LN2	LN	Roider et al	2071	117	2	1,71
rLN3	sc_LN3	LN	Roider et al	2816	194	9	4,64
LN4	sc_LN4	LN	Internally generated data	5247	625	20	3,20
T2	sc_tonsil1	tonsil	Steen et al	3301	168	10	5,95
Tonsil2	sc_tonsil2	tonsil	Internally generated data	6245	454	22	4,85
Tonsil3	sc_tonsil3	tonsil	Internally generated data	5624	300	16	5,33
Tonsil4	sc_tonsil4	tonsil	Internally generated data	6874	526	30	5,70

Supplementary table 8. Top 50 differentially expressed genes in MC09 scRNA-seq cluster.

p_val	avg_log2FC	pct.1	pct.2	p_val_adj	cluster	gene
7,214E-291	2,08633069	0,897	0,445	1,346E-286	MC09	LAG3
1,3224E-200	1,64064575	0,77	0,325	2,467E-196	MC09	PTMS
8,6759E-199	1,81694009	0,683	0,245	1,618E-194	MC09	HAVCR2
5,9397E-194	1,21629301	0,948	0,695	1,108E-189	MC09	CD27
3,0245E-178	2,25089612	0,566	0,18	5,642E-174	MC09	TNFRSF9
8,1914E-178	2,2236814	0,502	0,137	1,528E-173	MC09	LINC01943
7,6404E-171	0,99273752	0,988	0,951	1,425E-166	MC09	GAPDH
1,1677E-170	3,67516992	0,67	0,286	2,178E-166	MC09	CCL4L2
6,5481E-167	1,86941704	0,55	0,174	1,221E-162	MC09	FASLG
2,6221E-165	1,86890819	0,722	0,329	4,891E-161	MC09	BATF
7,0222E-161	1,5355773	0,863	0,565	1,31E-156	MC09	PRF1
7,4312E-157	2,11489678	0,489	0,146	1,386E-152	MC09	CADM1
4,3E-154	1,53108894	0,709	0,304	8,021E-150	MC09	IFNG
2,0122E-153	1,50698216	0,639	0,238	3,754E-149	MC09	PDCD1
9,1821E-147	1,16840832	0,949	0,842	1,713E-142	MC09	NKG7
4,1912E-139	2,02153616	0,474	0,152	7,818E-135	MC09	TIMD4
2,3418E-136	1,39213516	0,756	0,386	4,368E-132	MC09	CHI3L2
7,1508E-133	1,38374484	0,685	0,312	1,334E-128	MC09	CXCR6
9,6705E-133	1,33052244	0,854	0,583	1,804E-128	MC09	PKM
1,2058E-123	2,33911447	0,897	0,592	2,249E-119	MC09	CCL4
2,892E-123	1,10864388	0,91	0,678	5,395E-119	MC09	ITM2A
1,4788E-122	1,8577525	0,542	0,213	2,759E-118	MC09	CCL3
2,1262E-121	1,24426107	0,829	0,514	3,966E-117	MC09	TIGIT
4,9419E-118	2,83710396	0,274	0,057	9,219E-114	MC09	ASCL1
8,0982E-117	1,14823283	0,817	0,502	1,511E-112	MC09	PGAM1
1,422E-115	2,38986328	0,266	0,053	2,653E-111	MC09	ADTRP
7,2064E-113	1,69341943	0,375	0,104	1,344E-108	MC09	AC243829.4
2,1428E-110	1,18846817	0,703	0,358	3,997E-106	MC09	EOMES
2,541E-107	1,82063615	0,335	0,09	4,74E-103	MC09	HMOX1
4,9543E-104	1,04469814	0,787	0,503	9,242E-100	MC09	FKBP1A
4,1073E-103	3,29016918	0,277	0,068	7,662E-99	MC09	IL10
6,4176E-103	2,050991	0,313	0,082	1,1971E-98	MC09	SEMA4A
1,5643E-101	1,20551331	0,68	0,353	2,9181E-97	MC09	TNFRSF1B
6,4115E-101	1,22532402	0,686	0,362	1,196E-96	MC09	HLA-DMA
7,8406E-101	2,08069535	0,303	0,078	1,4626E-96	MC09	VCAM1
3,1245E-100	1,86250453	0,316	0,085	5,8285E-96	MC09	ADGRG1
4,0551E-99	1,0930697	0,717	0,395	7,5644E-95	MC09	SIRPG
7,44507E-98	1,36110413	0,505	0,204	1,3888E-93	MC09	SNAP47
8,97451E-98	2,31576275	0,254	0,058	1,6741E-93	MC09	SLC27A2
1,83487E-95	2,45966254	0,256	0,06	3,4228E-91	MC09	TNIP3
1,25352E-91	1,17415871	0,65	0,337	2,3383E-87	MC09	CD82
4,26916E-91	0,97807167	0,808	0,554	7,9637E-87	MC09	CTSC
6,53552E-91	1,22504165	0,614	0,306	1,2191E-86	MC09	GZMH
5,30418E-87	1,66725056	0,366	0,125	9,8944E-83	MC09	TSPAN17
5,35782E-87	1,89023279	0,578	0,293	9,9945E-83	MC09	GZMB
1,70319E-86	1,03181714	0,701	0,411	3,1771E-82	MC09	ANXA5
8,74137E-86	2,23570336	0,286	0,082	1,6306E-81	MC09	MIR155HG
7,3373E-84	1,05614611	0,674	0,38	1,3687E-79	MC09	PLEK
7,68948E-81	1,55934877	0,366	0,131	1,4344E-76	MC09	RGS3
